# Books and Authors

**Published:** 1909-10

**Authors:** 


### BOOKS AND AUTHORS.

**Handbook of Diseases of the Rectum.** By **Louis J. Hirschman,** lecturer on rectal surgery, Detroit College of Medicine. Octavo, pp. 374. St. Louis: C. V. Mosby Medical Publishing Co.

It is true, we believe, that the specialty of which this book treats is less represented in the literature of medicine than most others,—less written up in book form at least. To be sure there are many monographs published and publishing, and the American Procotologic Society, the proceedings of which we are publishing in part in the current issue of the JOURNAL, is doing much to improve and increase our knowledge on this subject.

The design of this book is to equip the general practitioner with adequate knowledge to diagnosticate and treat diseases of the anus and rectum as they more commonly are presented to him. The specialist will always have reserved to him the more difficult surgical cases, as a matter of course, but this book will do much, if it is carefully studied, to relieve him of anxiety concerning the simpler forms of ano-rectal disease.

Hirschman deals intelligently with his subject; his object is to make a handbook, in which the general practitioner might find information to aid him in the treatment of the diseases dealt with, and we think he has succeeded most admirably in accomplishing his purpose. The text is clear and direct, the illustrations are excellent, and the mechanical part of the book is all that could be desired.

Several of the chapters deserve special mention. The author is to be commended for the one on pruritus ani, sometimes a most obstinate condition to deal with, occasionally, indeed, resisting all methods of treatment short of surgical operations. The chapters on fistula, abscess, hemorrhoids, the author's technic, and his bloodless operation for certain forms of internal hemorrhoids all bespeak the quality of the author's work which we regard as of the best.

---

**Diet in Health and Disease.** By **Julius Friedenwald,** Professor of Gastroenterology in the College of Physicians and Surgeons, Baltimore, and **John Ruhrah,** M.D., Professor of Diseases of Children in the College of Physicians and Surgeons, Baltimore. Third edition. Octavo, pp. 765. Philadelphia and London: W. B. Saunders Co. (Price, $4.00.)

The favor accorded this work on its first appearance and in its second edition, has prompted the authors to bring out a new edition under complete revision. The book as originally prepared, was intended to meet the wants of both practitioner and student, as well as that of a manual for training schools. Nor have the authors in this edition departed from their original purpose but have merely elaborated it, keeping to the general idea of producing a practical handbook.

Some of the articles have been rewritten, notably those on milk and alcohol, while to others additions have been made. Some tables, also, have been added showing the caloric value of foods, and Winton's table appears showing the composition of diabetic foods, which is of great value. A short account, too, of the simpler methods employed to detect certain food adulterations and preservatives has been inserted. The book, therefore, may be regarded as reflecting the latest and best thought on the subject of which it treats.

---

**Treatment of the Diseases of Children.** By **Charles Gilmore Kerley,** Professor of Diseases of Children in the New York Polyclinic Medical School and Hospital. Second edition. Octavo, pp. 629. Philadelphia and London: W. B. Saunders Co. 1909. (Cloth, $5.00.)

In reviewing the first edition of this work, we expressed the opinion that it would take a high place amongst the literature of the diseases of children. The appearance of a second edition so soon after the first, would seem to indicate that our predictions were not erroneous. It is, most emphatically, a book for the general practitioner, one that he may keep at hand for every day consultation. Moreover, it is one that will give him confidence in his work among his little patients. In this respect, the second edition is even in advance of the first, having been strengthened here and there, and every weak point is now protected. The tables of drugs and drug dosage both for internal and external use, beginning on page 575 are most valuable. The index, comprising thirty-six pages is most complete,—one of the best,—increasing the worth of the book very much. We cannot speak too highly in praise of this most excellent work.

---

**Vaccine and Serum Therapy.** By **Edwin Henry Schorer,** Assistant Professor of Parasitology and Hygiene, University of Missouri. Octavo, pp. 131, illustrated. St. Louis: C. V. Mosby Co. (Price, $2.00.)

In these days of treatment by methods that are believed to have selective action in relation to cure, it is essential that we should know the facts, that everything bearing on vaccines and immune sera shall be set forth in accessible form, and in plain terms. This author seems to have enthusiasm on the subject; believes in the theories of immunity and in methods of vaccine and serum therapy. He accepts the opsonic theory of immunity, introduces the opsonic index and discourses upon the importance of opsonins in health and disease.

The author was taught the opsonic index technic given in his book by Dr. W. G. Ross, who in turn was for two years the pupil of Sir A. E. Wright, the original promulgator of the opsonic theory. A careful study of this book without doubt will equip both student and practitioner with the latest information on the subject.

**Human Physiology, an Elementary Textbook of Anatomy, Physiology and Hygiene.** By **John W. Ritchie,** Professor of Biology, at the College of William and Mary, Virginia. Illustrated by **Mary H. Wellman.** Yonkers-on-Hudson, N. Y.: World Book Company. 1909.

Elementary physiology, if taught at all, should be well taught; in order to teach it properly, the textbook used should be accurate. We have examined this book with some care and have convinced ourselves that Professor Ritchie has accomplished his purpose. One of the chief objections to teaching physiology in the primary schools has been slipshod methods and inaccurate books. With the introduction of such books as this, the latter objection will be removed. We trust the book will be adopted by the superintendents of education wherever it is deemed best to teach physiology in the primaries.

---

**Manual of Therapeutics** referring especially to the products of the Pharmaceutical and Biological Laboratories of Parke, Davis & Company. Detroit: Parke, Davis & Company. 1909.

This manual is a well-printed volume, handsomely bound in flexible leather, of about 650 pages. It is really an encyclopedia of useful information which the physician is sure to value, considering the convenient form in which it is presented. The first 38 pages speak for themselves; but we especially desire to direct attention to the therapeutic suggestions, on pages 39 to 96. The bulk of the book is devoted to materia medica, pages 97 to 632. The authors have striven here to place before the hardworking practitioner, in the most convenient form, a means of perceiving at a glance every available form of medication. By referring to the chapter on therapeutic suggestions and to the larger chapter on materia medica, he can see quickly just what drugs, or preparations of a given drug, are available.

It is our impression, after making careful examination of the book, that it will prove of vast usefulness to every practising physician and our readers will do well to order each a copy, particularly as by asking the publishers, one may be had without price.

---

**The Practical Medicine Series.** Ten volumes. Issued under the general editorial charge of **Gustavus P. Head,** M.D., Professor of Laryngology and Rhinology in the Chicago Post-Graduate Medical School. Volume III. Eye, ear, nose and throat. Edited by **Casey A. Wood, Albert H. Andrews, Gustavus P. Head.** Series 1909. Chicago: The Year Book Publishers. (Price, $1.25; entire series, $10.00.)

The advances in ophthalmology during 1908 are abstracted for this work by Casey A. Wood, of Chicago, who has performed a similar service for the previous volumes of the series relating to this topic. The ophthalmo-tuberculin reaction, having been tested, is now and here given its well defined place. It is well, too, that the relationship between general medicine and ophthalmology is better understood and is given recognition. The abstracts relating to the eye occupy about half of the book.

The ear is presented in abstract by Albert H. Andrews, and the mastoid, as well as the middle ear, affords interesting material, showing all important improvements of the year.

The nose and throat literature of the year is compiled by the general editor, Gustavus P. Head. The accessory cavities, as expected, come in for a full share of importance, and the abstractor has shown good judgement in selecting his material.

